# Supplementary material for: HPLC, FTIR and GC-MS Analyses of Thymus vulgaris Phytochemicals Executing In Vitro and In Vivo Biological Activities and Effects on COX-1, COX-2 and Gastric Cancer Genes Computationally
Source: Molecules. 2022 Dec 3;27(23):8512. doi: 10.3390/molecules27238512 (PMC9736827; doi:10.3390/molecules27238512)
Supplement: Supplementary file 1 [file molecules-27-08512-s001.zip › Figure S1.pdf]

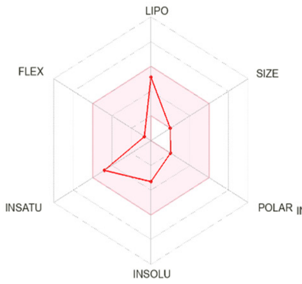

Carvacrol

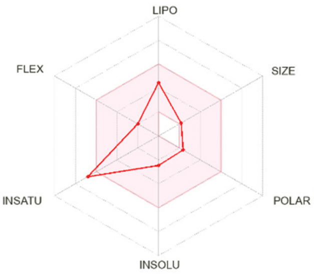

Eugenol

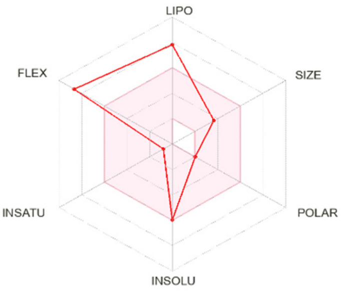

Phytol

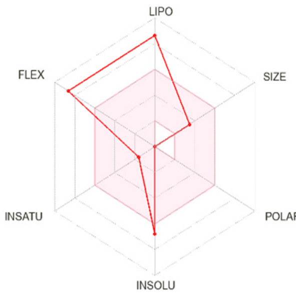

Neophytadiene

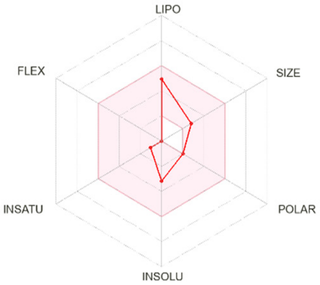

Spathulenol

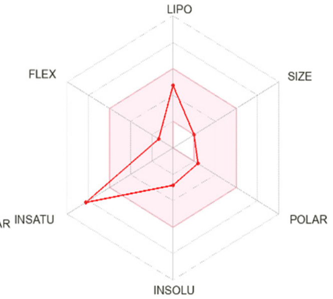

Methoxy-4-vinylphenol

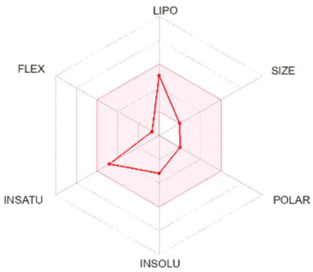

Thymol

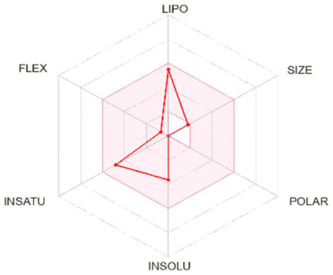

p-Cymene

Figure S1. Radar plot of bioactive compounds.
