# Supplementary material for: HPLC, FTIR and GC-MS Analyses of Thymus vulgaris Phytochemicals Executing In Vitro and In Vivo Biological Activities and Effects on COX-1, COX-2 and Gastric Cancer Genes Computationally
Source: Molecules. 2022 Dec 3;27(23):8512. doi: 10.3390/molecules27238512 (PMC9736827; doi:10.3390/molecules27238512)
Supplement: Supplementary file 1 [file molecules-27-08512-s001.zip › Table S1.pdf]

Table S1. ADME analysis of bioactive compounds derived from GC-MS analysis.

**Lipophilicity**	**Methoxy-4-vinylphenol**	**Eugenol**	**P-cymene**	**Carvacrol**	**Thymol**	**Phytol**	**Neophytadiene**	**Spathulenol**
Log Po/w (iLOGP)	2.25	2.37	2.51	2.24	2.32	4.85	5.05	3.04
Log Po/w (XLOGP3)	3.49	2.27	4.10	3.49	3.30	8.19	9.62	3.11
Log Po/w (WLOGP)	2.82	2.13	3.12	2.82	2.82	6.36	7.17	3.39
Log Po/w (MLOGP)	2.76	2.01	4.47	2.76	2.76	5.25	6.21	3.67
Log Po/w (SILICOS-IT)	2.79	2.48	3.29	2.79	2.79	6.57	7.30	3.27
Consensus Log Po/w	2.82	2.25	3.50	2.81	2.80	6.25	7.27	3.30
**Water solubility**								
Class	Soluble	Soluble	Soluble	Soluble	Soluble	Moderately soluble	Poorly soluble	Soluble
Log S (ESOL)	−3.31	−2.460	−3.630	−3.31	−3.190	−5.98	−6.77	−3.17
Solubility (mg/ml)	0.740	0.569	0.031	0.740	0.097	0.310	0.474	0.151
Log S (Ali)	−3.60	−2.530	−3.810	−3.60	−3.400	−8.47	−9.53	−3.20
Solubility (mg/ml)	0.379	0.490	0.021	0.379	0.060	0.994	0.815	0.138
Log S (SILICOS-IT)	−3.01	−2.790	−3.570	−3.01	−3.010	−5.51	−6.11	−2.96
Solubility (mg/ml)	0.146	0.265	0.068	0.146	0.146	0.906	0.218	0.239
**Pharmacokinetics**								
GI absorption	High	Low	High	High	Low	High	Low	High
BBB permeant	Yes	Yes	Yes	Yes	No	Yes	No	Yes
P-gp substrate	No	No	No	No	Yes	No	Yes	No
CYP1A2 inhibitor	Yes	No	Yes	Yes	No	Yes	No	No
CYP2C19 inhibitor	No	No	No	No	No	No	No	Yes
CYP2C9 inhibitor	No	No	No	No	Yes	No	Yes	No
CYP2D6 inhibitor	No	Yes	No	No	No	No	No	No
CYP3A4 inhibitor	No	No	No	No	No	No	No	No
Log K_p (*cm/s*)	−5.69	−4.21	−5.69	−4.87	−2.29	−4.74	−1.17	−5.44
**Drug likeness**								
Lipinski	Yes	Yes	Yes	Yes	Yes	Yes	Yes	Yes
Ghose	No	No	Yes	No	No	No	No	Yes
Veber	Yes	Yes	Yes	Yes	Yes	No	No	Yes
Egan	Yes	Yes	Yes	Yes	Yes	No	No	Yes
Muegge	No	No	No	No	No	No	No	No
Bioavailability score	0.55	0.55	0.55	0.55	0.55	0.55	0.55	0.55
